# A transfer function approach to measuring cell inheritance

**DOI:** 10.1186/1752-0509-5-31

**Published:** 2011-02-22

**Authors:** Paul Rees, M Rowan Brown, Huw D Summers, Mark D Holton, Rachel J Errington, Sally C Chappell, Paul J Smith

**Affiliations:** 1 Centre for Nanohealth, School of Engineering, Swansea University, Swansea, SA2 8PP, UK; 2 School of Medicine, Cardiff University, Heath Park, Cardiff, CF14 4XN, UK

## Abstract

**Background:**

The inheritance of cellular material between parent and daughter cells during mitosis is highly influential in defining the properties of the cell and therefore the population lineage. This is of particular relevance when studying cell population evolution to assess the impact of a disease or the perturbation due to a drug treatment. The usual technique to investigate inheritance is to use time-lapse microscopy with an appropriate biological marker, however, this is time consuming and the number of inheritance events captured are too low to be statistically meaningful.

**Results:**

Here we demonstrate the use of a high throughput fluorescence measurement technique e.g. flow cytometry, to measure the fluorescence from quantum dot markers which can be used to target particular cellular sites. By relating, the fluorescence intensity measured between two time intervals to a transfer function we are able to deconvolve the inheritance of cellular material during mitosis. To demonstrate our methodology we use CdTe/ZnS quantum dots to measure the ratio of endosomes inherited by the two daughter cells during mitosis in the U2-OS, human osteoscarcoma cell line. The ratio determined is in excellent agreement with results obtained previously using a more complex and computational intensive bespoke stochastic model.

**Conclusions:**

The use of a transfer function approach allows us to utilise high throughput measurement of large cell populations to derive statistically relevant measurements of the inheritance of cellular material. This approach can be used to measure the inheritance of organelles, proteins etc. and also particles introduced to cells for drug delivery.

## Background

The function of an organism is determined by the evolution of a cell population all descended from a single progenitor cell. The behaviour of individual cells is thus determined not only by their environment but also by the composition of cellular material inherited from the parent cell during mitosis. Therefore, the segregation of cellular material during mitosis is critical in determining the fate of the daughter cells. Previously, it was assumed that mitotic partitioning of cellular material was equal between daughter cells [1], however more recent studies have shown that certain cell components partition asymmetrically at mitosis [2-5] and there is a growing realisation that division asymmetry is a fundamental property of biological cells. For example, it has been demonstrated that proteins destined for proteasomal degradation within aggresomes are preferentially inherited by one daughter and it has been suggested that the generation of different daughter cells promotes long term lineage survival [6]. Stem cell differentiation has also been linked to asymmetric inheritance of endosome function in daughter cells [2,7].

Whilst division asymmetry is now clearly recognised, it has been difficult to accurately quantify as most studies rely on time-lapse microscopy, which can provide measurements on a limited number of cells. There is a need therefore for statistically relevant measures of inheritance quantifying the inter-generational transformation across a cell population. In previous studies, we have demonstrated that through flow cytometric analysis of colloidal quantum dot (QD) fluorescence the asymmetric inheritance of endosomes can be accurately quantified, based on measurements from sets of > 10^4 ^cells [5]. This technique employed a stochastic cell model of all individual cells within a population to predict the generational redistribution of the QD-loaded endosome fluorescence over time. The model parameters were optimised, via an evolutionary algorithm, to maximise correlation between the fluorescent histograms generated numerically to that experimentally measured. This process predicts an asymmetric redistribution of QDs across the population [8] as opposed to the intuitive guess of symmetric partitioning. While this approach proved a robust method for analysing cellular inheritance, the algorithms employed to predict the evolvution of the *in-silico *population are numerically bespoke requiring an in-depth knowledge of the biological principles and processes involved during the complex cell-cycle.

The purpose of this paper is to outline a method for assessing the inheritance of cellular material using high throughput fluorescence measurements combined with simple and more accessible models derived from systems theory. The focus of systems biology is usually on understanding the complex interactions which occur between the components of a biological system e.g. the protein interaction networks driving a specific cellular function or response. By identifying the relevant components within the complex system a host of mathematical tools can be used to attempt to identify the nature of the mechanisms between them. Here we take inspiration from traditional systems engineering to take an alternative viewpoint that concentrates on the dynamics of the whole system (cellular population evolution) through relation of initial and final states of that system by a transfer function. A transfer function is simply a mathematical expression which relates the input (function) and the output (function) of a (non-)linear system. It must the formulated to include the physical (or biological) processes involved in the system which modifies the variable or quantity that is being measured. We use the transfer function approach to encapsulate and transform the stochastic population dynamics of the stochastic cell cycle model into a continuous form. This entails a significant simplification of the complex processes associated with the biological system processes, as they are now described purely phenomenologically through their influence on the evolving cellular state. However we will demonstrate that appropriately designed experiments coupled with simple mathematical models of the transfer function can accurately elucidate biological function and provide a generalised analysis method that delivers relevant information on the system without requiring *a-priori *knowledge of sub-cellular processes. Casting the problem in terms of a transfer function between an initial and final state-function opens up the possibility of using system analysis such as convolution theory to deconvolve cell inheritance information from flow measurements.

**Figure 1:**
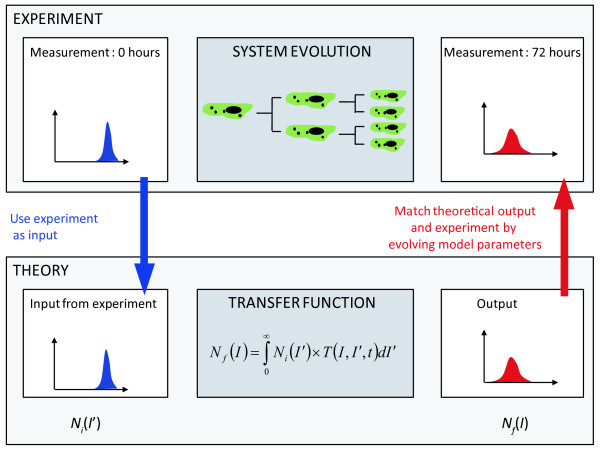
**Schematic representation of the transfer function approach to extract information from a cell population**. Fluorescence intensity of the cell population measured at 0 hours is used as the input for a transfer function which generates an output that is compared with a fluorescence intensity measured at a time t later. Genetic algorithms are used to optimise the variables of the transfer function to best fit the experimental data.

A schematic of our approach is given in figure 1. The quantum dot fluorescent intensity histogram measured at *t = 0 *hours via the flow cytometer provides the input function. The cells are then incubated for a set amount of time during which the cells within the population evolve through their cell cycles until mitosis when the labelled cellular material (endsome encapsulated QDs) is redistributed to the daughter cells. A second measurement of the fluorescence then provides the output function for the analysis. We derive a simple transfer function which acts on the input function and the resultant function is compared with the output function obtained from the second measurement. The parameters of the transfer function can then be optimised to obtain the best fit between experiment and the output function. To demonstrate the concept we use engineering system theory to measure the partitioning of endosomes from parent to daughter cells during mitosis. By using flow cytometry we are able to measure the fluorescence of many cells (>10^4^) in a population which allows us to derive a large enough data set to be statistically relevant.

## Results and Discussion

First we define the transfer function which effectively delineates the process that we are able to quantify. We assume that the labelled cellular material is conserved as the cell undergoes mitosis and is redistributed between the two progeny, and introduce the ratio *α *(which is a real number between 0 and 1) to represent the proportion of labelled material inherited by each daughter cell as *α:(1-α)*. The consequence of applying this concept to the cell population is a high degree of variation of inherited cellular material between individual mitotic events. However, providing we consider a large number of bifurcating events (as we can through flow cytometry based measurement) we can describe the partitioning ratios of all the cells as a continuous Gaussian probability distribution function with a mean *α *and standard deviation *Δα*. Therefore, the probability of a cell undergoing mitosis with a splitting ratio of *x *is given by:

(1) $$P\left(x\right)=\frac{1}{2\sqrt{2}\pi \Delta \alpha }\left\{e^{-{\left(\frac{x-\alpha }{\Delta \alpha }\right)}^{2}}+e^{-{\left(\frac{x-\left[1-\alpha \right]}{\Delta \alpha }\right)}^{2}}\right\}$$

The aim of the analysis is to effectively determine this distribution i.e determine *α *and *Δα *for the mitosis events within a cell population. The technique does not measure the inheritance for a single cell, but rather a distribution of inheritance based on all cells within the population. The assumption that we are dealing with large cell numbers allows us to use the continuous Gaussian distribution to represent the random variation of the inheritance around the mean value.

**Figure 2:**
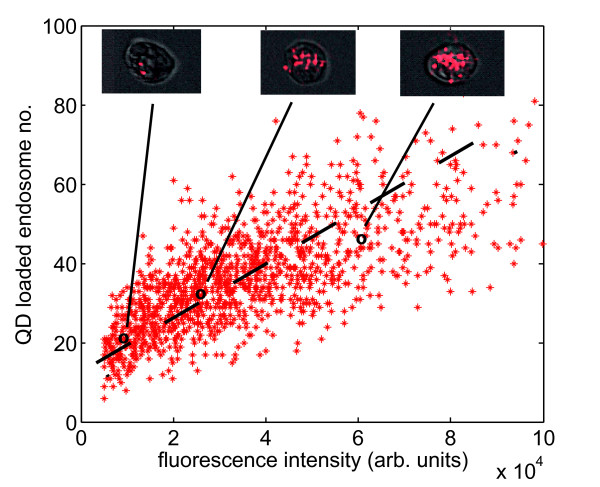
**Number of quantum dot loaded endosomes versus the fluorescence intensity measured for that cell**. The number of quantum dot loaded endosomes is plotted versus the fluorescence intensity measured by flow cytometry for that particular cell. The linear relationship between the measured fluorescence and the number of organelles labelled is a requirement for the transfer function approach. The insets show typical examples of the cell images used to construct the data by the imaging cytometer: the quantum dot loaded endosomes are clearly visible.

To demonstrate our concept Qtracker^® ^705 nm *CdTe/ZnS *(Invitrogen) quantum dots are loaded into the endosomal compartments of U2-OS cells and subsequently the fluorescence intensity of each cell is measured using an imaging flow cytometer; i.e. the measured parameter is total fluorescence intensity and the inherited cellular material are the QD loaded endosomes. This approach clearly requires that the fluorescence intensity measure be proportional to labelled cellular material investigated. To establish the correlation between cell fluorescence and number of QD-endosomes an imaging cytometer was used to provide direct visualisation of the endosome number corresponding to specific values of total cell fluorescence. The resulting correlation is shown in figure 2 confirming that indeed the cell fluorescence can be employed as an accurate surrogate for endosome number i.e. the endosome inheritance patterns can be tracked via fluorescence intensity measurements.

**Figure 3:**
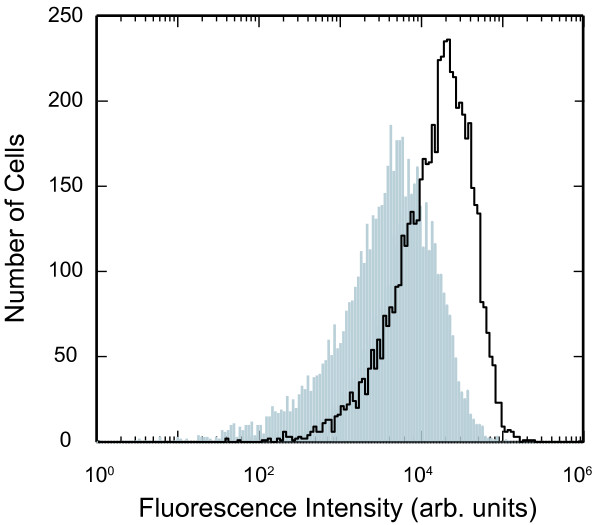
**Number of cells versus the fluorescence intensity histogram**. The first fluorescence histogram measured at time = 0 using a flow cytometer is shown as the solid line outline. The second measurement 19 hours later is shown as the grey histogram.

Typical results are shown in figure 3 as a fluorescence intensity histogram, *N*_*i*_*(I) *where the number of cells is plotted as a function of the total fluorescence intensity within the cell. Cells are then incubated for a predetermined time, *t *(relative to the first measurement) and a second measurement is performed, again shown in figure 3 as the histogram *N*_*f*_*(I)*. Clearly the fluorescence intensity of the cells is decreasing; the fluorescence stability of the QDs within the cellular environment has been previously established [5] and therefore the decreasing intensity indicates that the number of QD-labelled endosomes per cell reduces through dilution due to mitotic events.

Using the terms of systems analysis we would like to derive a transfer function *T(t) *which acts on the initial state *N*_*i*_*(I) *to produce the final state *N*_*f*_*(I)*: the intensity histogram measured at time *t*. In implementing this analysis, *t *is chosen such that it encompasses a single round of cell division so that the initial and final states correspond to parent and daughter cells respectively. We note that the transfer function defines the systemic event we are measuring (fluorescent intensity partition between daughter cells) not any particular biological process. However, these measurements and therefore the transfer function allows us to deconvolve useful biological information, such as the partitioning of the endosomes between two daughter cells. If we now consider the cells in the population with a measured fluorescence intensity, *I'*, then if all these cells undergo a mitosis event then the proportion of the daughter cells having a fluorescence intensity, *I*, would be given by *P(I/I')*. The division by *I' *normalises by the parental value to give a range between zero and one for use in equation 1. By considering all cells in the population, we can relate the two measured intensity histograms *N*_*i*_*(I) *and *N*_*f*_*(I) *by:

(2) $$N_{f}\left(I\right)=\int_{0}^{\infty }N_{i}\left(I'\right)\times T\left(I,I',t\right)dI'$$

and *T*, is the transfer function is given by:

(3) $$T\left(I,I',t\right)=f_{t}P\left(\frac{I}{I'}\right)+\left(1-f_{t}\right)\delta \left(I=I'\right)$$

where *f*_*t *_is the fraction of cells, having undergone mitosis during *t *and *δ *is the Dirac delta function.

We note that the implementation of the convolution integral in equation 2, requires a summation over the fluorescence intensity and therefore it is worth discussing how the intensity data is prepared to provide the input function *N*_*i*_*(I) *for the transfer function *T(I,I',t)*. The high-throughput fluorescence imaging technique would need to measure the total intensity per cell, however, in most flow cytometers the fluorescence intensity of the cell is measured using a logarithmic amplifier in order to maximise the dynamic range of measurement and subsequently the output is in the form of a histogram with logarithmic bin spacing [9]. This can easily be dealt with by either converting to a linear intensity histogram or applying the transfer function with an integral summed over variable intensity width appropriate for the logarithm scale. While flow cytometry is an ideal technique for implementation of this technique, we also note instruments such as high-throughput microscopes, imaging cytometers etc. can also be used to provide the average fluorescence intensity of each cell.

The first term on the right hand side of equation 3 describes the probability of a change in fluorescence intensity from *I' *to *I *given that *f*_*t *_is the fraction of cells having undergone mitosis in the time *t when the second fluorescence intensity measurement is performed*. The second term represents the cells which have not undergone mitosis and therefore their fluorescent intensity remains unchanged. Now provided we can determine *f*_*t *_we are in a position to apply equation 3 to the first measurement and optimise parameters α and Δα to obtain the best fit with the fluorescent intensity measurement at time *t*. There are several approaches we can take to determine *f*_*t*_. The simplest approach is to use the variable as a fitting parameter which gives three unknown variables within the transfer function to optimise. This does not cause any problems for any fitting algorithm employed as the cells which have not undergone mitosis have the highest intensity (while the daughter cells have a lower intensity) and therefore the shape of the intensity profile at high values is highly sensitive to the value of *f*_*t *_while remains insensitive to the values of α and Δα.

**Figure 4:**
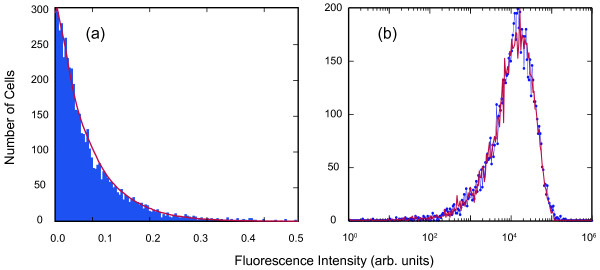
**The number of cells versus the fluorescence intensity**. Figure (a) shows a histogram depicting the number of cells as a function of the intensity fluorescence normalised to the peak intensity measured. The blue histogram represents the experimental values (the grey histogram in figure 3) measured after 19 hours and the red curve represents the values obtained using the transfer function applied to the experimental measurement at time 0. Figure (b) shows the same information using the same colour scheme however it is now plotted using a logarithm intensity scale as generated by a flow cytometer.

In figure 4 we have applied the transfer function to the initial fluorescence histogram and optimised the parameters to best fit the second fluorescence histogram measured 19 hours later. We use the same evolutionary algorithms to obtain the best parameter fits as described in previous work, which validated this approach when fitting stochastic cell cycle models to flow data [5]. The fit shown in figure 4 was generated with a value of *α *= 0.71 and *Δα *= 0.11 and the fraction of cells determined to have undergone mitosis during this 19 hour period was, *f*_*t *_= 0.98. The fit gives a p value from a *t*-test of approximately 0.975 between the experimental histogram and the histogram derived using the transfer function which confirms the simple assumptions of the partitioning process at mitosis. The average intermitotic time of the U2-OS cells was previously measured to be approximately 20 hours [5] which also suggests the majority of the cells would have undergone mitosis in 19 hours. The transfer function parameters are also in excellent agreement with the splitting ratio distribution (α and Δα) obtained using our more complex analysis using stochastic cell cycle models [8].

Whilst the dividing fraction, *f*_*t *_is obtained through the fitting procedure it is also possible to deduce its magnitude from experimental measurements. Irrespective of the inheritance ratio the *average *value of fluorescence intensity of the two daughter cells is going to be half that of the parent cell. Therefore we can write the following:

(4) $$\langle I_{t}\rangle =\left(1-f_{t}\right)\langle I_{0}\rangle +f_{t}\frac{\langle I_{0}\rangle }{2}$$

where *I*_*0 *_and *I*_*t *_are the mean intensity of the cell population measured at time *0 *and *t *respectively, obtained from the experimental flow histograms. This is of course only valid if we measure the fluorescence intensity from the same number of cells in each measurement. Using the data from the fluorescence histograms in figure 3 we obtain a value *f*_*t *_= 0.969 which is excellent agreement with the value determined using the fitting procedure. While this gives us confidence in the fitting procedure used, it is also possible to use this experimental value in the transfer function and therefore just fit for values of *α *and *Δα*.

We note most of the cells within the population would have undergone mitosis during the time duration chosen between the measurements. When designing this type of experiment it is important to maximise the number of mitosis events which helps increase the accuracy of the splitting parameters determined by the fitting procedure. However if the time interval between the two fluorescence intensity measurements becomes too long then the daughter cells themselves would start to undergo mitosis which adds another layer of complexity to the analysis. The measure of the fraction of cells that have undergone mitosis determined using equation 4 can provide a useful tool (if the intermitotic time of the cell line is unknown) in deciding the time interval between the two fluorescence measurements. The relative number of cells in each generation can be calculated for example using branching theory [10] however this requires a knowledge of the cell intermitotic time. The issue of the time between measurements also raises a further consideration for the planning of such an experiment. The analysis can only measure the inheritance of labelled cellular material and therefore the inheritance of any new cellular material which develops during the cell cycle cannot be elucidated upon.

As discussed earlier the method describe so far relies on the time period of the experiment to be sufficiently short so that no cell in the population divides twice. This is simply due to the way we have defined our transfer function (equation 3) in terms of cells that have split once and those that have not undergone mitosis. If an experiment necessitated a time period where several generations of cells might be observed then the analysis is perfectly valid providing the transfer function in equation3 included an extra term for each generation. For example, if 3 generations were to be observed an extra term would be included to describe the fraction of cells which had 'undergone two mitosis events'. The probability distribution associated with the cells would be a modified form of equation 1 which would now include 4 intensity partitioning Gaussian distributions for the 4 cells (originating from the one progenitor cell) in the third generation.

While for this study we have used fluorescent quantum dot markers to label the organelles, this protocol can be used with any other fluorescent dye providing a couple of criteria are met. Firstly the dye must fluoresce with intensity proportional to the quantity of organelles to be measured. Secondly the intensity of the dye must remain stable for the duration of the experiment. Also if the dye fluorescence intensity does deteriorate during the experiment by a quantifiable amount then this can be compensated for in the transfer function as a correction to the magnitude of the Gaussian distributions in equation 1.

## Conclusions

In summary, we have presented a transfer function method for determining the inheritance of cellular material; the partitioning of endosomes loaded with colloidal quantum dots between daughter cells has been measured using flow cytometry. The use of a transfer function methodology provides a generic framework that is suitable to a wide range of cytological analyses. In particular it relates transformative states of the cell and in doing so provides quantitative information on the system evolution without the need for *a-priori *knowledge of the system components such as molecular networks or gene expression profiles. This whole-system approach is grounded in experimental observation, based as it is on the mathematical linking of two observational cell descriptor functions. In this work we compare whole cell parameters over a large population but the approach is equally valid for analysis of the evolution of a single cell captured using fluorescence microscopy. Cellular inheritance provides an ideal test system for the use of transfer functions as bifurcations at cell division give clearly marked events in the system history. Furthermore, the studies reported here can easily be extended to measure the inheritance of other cellular organelles [11,12] or to describe the differing fate of daughter cells [7]. More subtle system evolution within a single cell generation could also be tracked for instance cell-cycle progression through cyclin-based fluorescent reporters [13] or cellular metabolism via measurements of fluorescence lifetime [14].

## Methods

### QD loading and preparation of cells

Human osteosarcoma cell populations (U-2 OS cell line, ATCC HTB-96) were maintained in McCoy's 5a medium supplemented with 10% foetal calf serum (FCS), 1 mM glutamine, and antibiotics and incubated at 37°C in an atmosphere of 5% CO2 in air. The cells were loaded with commercially available targeted nanocrystals using the Qtracker^® ^705 (QTracker705) Cell Labelling Kit (4 nM) (Invitrogen (Q25061MP). The QTracker system uses a proprietary peptide targeting molecule, attached to the dot surface to enable receptor mediated endocytosis. Compared to untargeted QDots this produces a rapid cellular uptake (loading rate time of ~ 15 minutes compared to 4 hours for untargeted, carboxyl coated QDs) [15]. The internalization of the QDots into discrete vesicles has been confirmed by confocal microscopy. The nature of the vesicle i.e. endosome or lysosome has not been established and will change over time, however this does not affect the division analysis as this considers numbers of labeled vesicles and is independent of their biological identity. The reagents in the Qtracker^® ^705 Cell Labelling Kit use a custom targeting peptide (9-arginine peptide) to deliver near-infrared-fluorescent nanocrystals into the cytoplasm of live cells via the endosomal pathway. Qtracker reagent A and B were premixed and then incubated for 5 mins at room temperature. 1 ml of fresh full growth media was added to the tube and vortexed for 30 seconds. This labelling solution was then added to each well of the cells and incubated for 1 hour at 37°C after which they were washed twice with fresh media and split into two flasks. To prepare for flow analysis samples were incubated in FACS Buffer (PBS/0.2% BSA/0.05% Sodium Azide) for 30 minutes before re-suspending in 200 μl of PBS and placed in a refrigerator at 4°C until later data acquisition on a flow cytometer. One sample was analysed using the flow cytometer while the second flask was incubated at 37°C for 19 hours and then analysed.

### Imaging cytometry

Cell images were acquired using an Imagestream100 Cell Analyser (Amnis Corporation, Seattle). A 488 nm wavelength laser was used to excite QD fluorescence which was collected via the 660-735 nm spectral detection channel. 10^4 ^cells were imaged for each sample and analyzed using the manufacturer's software. Gating of the acquired data to ensure focused images of viable cells reduced the analysed population to 5,000 cells. The system and peak image analysis algorithms were used to identify intensity clusters and calculate their number assuming a discrimination level of intensity at twice the intensity of the background. Spot counting accuracy was confirmed by manual verification of confocal images. The number of cells per ml within the gated population was also determined using the Imagestream100, which continuously runs speed calibration beads to provide absolute calibration of fluid flow rates.

## Authors' contributions

PR, MRB and HS wrote the paper with contribution by all authors. PR, HS, MRB, RE and PJS conceived of the study, MH and SCC performed the flow experiments and PR and MRB performed the data analysis. All authors read and approved the final manuscript.
